# The need for geriatric scales in glioblastoma

**DOI:** 10.18632/aging.203370

**Published:** 2021-07-23

**Authors:** Marta Domenech, Ainhoa Hernandez, Carmen Balana

**Affiliations:** 1 Applied Research Group in Oncology, Institute for Health Science Research Germans Trias I Pujol, Badalona, Spain

**Keywords:** glioblastoma, temozolomide, extended, age, survival

Glioblastoma patients older than 50 have long been considered to have a poor prognosis, although molecular alterations are now known to attenuate the impact of age on the diagnosis and prognosis of gliomas [1]. Since the pivotal trial in 2005, the gold standard of treatment for glioblastoma has been surgery followed by radiation therapy with concomitant and adjuvant temozolomide. However, the benefit of this regimen decreases with age, especially after 60 years, and in fact, few patients older than 65 were included in the original trial [2]. Unfortunately, median age at diagnosis of glioblastoma is 65 [3], so approximately 50% of patients are not candidates for the standard treatment. As a result, several randomized trials have led to other treatment options being proposed for elderly patients, such as treatment with temozolomide alone in patients with MGMT promoter methylation, irradiation alone in patients without MGMT methylation [4,5], and hypofractionated irradiation together with concomitant and adjuvant temozolomide in patients older than 65 with MGMT methylation [1,6]. Although geriatric scales have been applied to patients with other tumors, this has only rarely been the case in glioblastoma.

**Figure 1:**
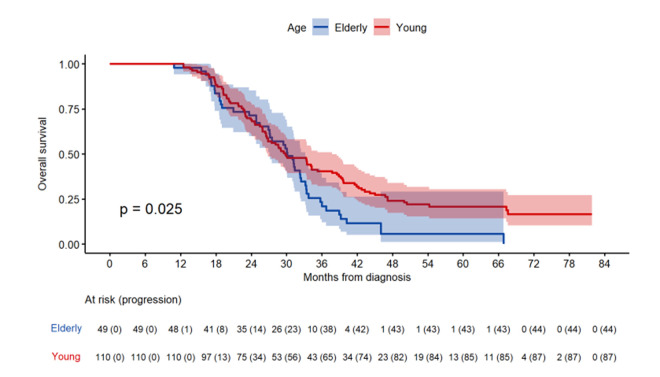
Overall Survival from diagnosis depending of group of age in the GEINO-14 trial.

The GEINO 14 trial [7] explored the role of extending treatment to 12 cycles of adjuvant temozolomide in patients who had already received the standard six cycles. Patients who remained progression-free after the first six cycles were randomized to continue for a further six cycles or to stop temozolomide. Importantly, older age was not considered an exclusion criterium in this trial. The results showed no difference in progression-free survival (PFS) or overall survival (OS) between the two treatment arms [7]. When we reanalyzed our data according to patient age, 49 patients were classified as elderly (>65; median age, 70; range, 66-83) and 110 as young (≤65 years; median age, 55; range, 29-65). Twenty-six elderly patients (53.1%) were randomized to the stop arm and 23 (46.9%) to the continuing arm (P=0.60). Twenty-eight (57.1%) had MGMT methylation. MGMT methylation status, Karnofsky performance status and measurable disease at inclusion were similar between elderly and young patients. Only one patient (2%) in the elderly group had IDH1 mutations, compared to eight (7.3%) in the young group. Median OS from randomization was similar in both groups (elderly, 20.4 months; young, 20 months; P=0.02), but long survival (>30 months) from diagnosis was attained by 51% of elderly and 47.8% of young patients (P=0.02) (Figure 1). Among all patients in the continuing arm, the median number of extended temozolomide cycles was six, and 75% of patients received all six additional cycles, although 25% of elderly patients in the continuing arm did not receive any additional cycle. Among elderly patients, there were no differences in PFS (P=0.16) or OS (P=0.58) according to treatment arm. In fact, the multivariate analysis of all patients identified only MGMT status (PFS, P<0.003; OS, P<0.001) and the presence of measurable disease (PFS, P=0.017; OS, P=0.005) – but not age (PFS, P=0.76; OS, P=0.09) or treatment arm (PFS, P=0.89; OS, P=0.63) – as independent prognostic variables.

These findings suggest that there is no benefit for continuing temozolomide longer than six cycles for elderly patients once they have received the standard six cycles of temozolomide. Importantly, they also highlight the fact that elderly patients can attain a similar survival benefit to that of younger patients provided they are fit enough to receive standard chemoradiation. Moreover, the elderly may attain long survival with the standard treatment. We can therefore suggest that the selection of the optimal treatment for elderly patients should not be based solely on age.

Age is certainly an important factor in neuro-oncology given its association with cognitive impairment, brain vasculopathy, and other associated organic pathologies. In addition, accompanying social factors, such as isolation and lack of family support, can impact in a disease like glioblastoma that frequently ends with a loss of the capacity for self-care and decision-making. However, age is not the only critical factor. To date, unfortunately, none of the trials conducted in elderly glioblastoma patients have included geriatric scales like those that are already being applied in the treatment of other cancers. Nevertheless, a recent retrospective study found significant differences in OS when elderly glioblastoma patients were stratified according to a geriatric scale and classified as fit, vulnerable, or frail [8]. The scales used for other tumors would need to be readjusted for brain tumors and include variables related to neurological function, but once readjusted, they would certainly be useful in determining the best therapeutic option for these patients – not only as regards post-operative treatment but also when deciding on the first neurosurgical approach.
